# Deep learning reveals 3D atherosclerotic plaque distribution and composition

**DOI:** 10.1038/s41598-020-78632-4

**Published:** 2020-12-09

**Authors:** Vanessa Isabell Jurtz, Grethe Skovbjerg, Casper Gravesen Salinas, Urmas Roostalu, Louise Pedersen, Jacob Hecksher-Sørensen, Bidda Rolin, Michael Nyberg, Martijn van de Bunt, Camilla Ingvorsen

**Affiliations:** 1 Novo Nordisk A/S, Novo Nordisk Park, 2760 Maaloev, Denmark; 2 Gubra, 2970 Hoersholm, Denmark; 3 University of Copenhagen, 1017 Copenhagen, Denmark; 4 Present Address: Gubra, 2970 Hoersholm, Denmark

**Keywords:** Computational biology and bioinformatics, Image processing, Cardiovascular biology

## Abstract

Complications of atherosclerosis are the leading cause of morbidity and mortality worldwide. Various genetically modified mouse models are used to investigate disease trajectory with classical histology, currently the preferred methodology to elucidate plaque composition. Here, we show the strength of light-sheet fluorescence microscopy combined with deep learning image analysis for characterising and quantifying plaque burden and composition in whole aorta specimens. 3D imaging is a non-destructive method that requires minimal ex vivo handling and can be up-scaled to large sample sizes. Combined with deep learning, atherosclerotic plaque in mice can be identified without any ex vivo staining due to the autofluorescent nature of the tissue. The aorta and its branches can subsequently be segmented to determine how anatomical position affects plaque composition and progression. Here, we find the highest plaque accumulation in the aortic arch and brachiocephalic artery. Simultaneously, aortas can be stained for markers of interest (for example the pan immune cell marker CD45) and quantified. In ApoE−/− mice we observe that levels of CD45 reach a plateau after which increases in plaque volume no longer correlate to immune cell infiltration. All underlying code is made publicly available to ease adaption of the method.

## Introduction

Complications of atherosclerosis remain the leading cause of morbidity and mortality worldwide, accounting for more than 18 million deaths each year^1^. It is a chronic inflammatory disease of large and medium-sized arteries characterized by accumulation of lipids, de-differentiated smooth muscle cells and leukocytes into the artery wall leading to the formation of atherosclerotic plaque. When the plaque advances, the plaque surface is covered by a fibrous cap and within the center of the plaque a necrotic core develops, consisting of lipids, cholesterol crystals and necrotic cells^2,3^. Clinical symptoms, such as myocardial infarction and stroke, typically arise from physical disruption of the fibrous cap. This exposes the coagulant interior into the lumen, eventually leading to thrombosis^4^.

In preclinical mouse studies, histology represents the golden standard for ex vivo characterization of atherosclerotic plaques^5^. This methodology provides the necessary resolution to analyse both the composition and morphology of atherosclerotic blood vessels. However, stereological sampling and histological analysis are time consuming and technically challenging, which makes the methodology inconvenient for large studies^5,6^. *En face* lipid staining of aorta represents a rapid way to visualize plaques in mouse models, but lacks volumetric information of plaques and resolution to make conclusions about plaque composition^7^.

Light-sheet fluorescence microscopy (LSFM) combined with whole-mount tissue staining and clearing enables 3D imaging of entire tissues at cellular resolution^8–15^. LSFM is a non-destructive imaging technique that makes paraffin-embedding and sectioning unnecessary, requiring little hands-on sample preparation time and offering great scaling capability (Supplemental Tables 1, 2). The technology has been applied to image parts of the cardiovascular system before^13,16–18^ Becher et al. have recently shown that this imaging technology can successfully be applied to detect plaque in brachiocephalic, carotid and radial arteries^19^. However, 3D light sheet imaging generates large data volumes that pose unique and so far unanswered challenges for rapid image analysis in particular in preclinical drug discovery studies.

Here, we present a fully automated workflow for 3D characterization and quantification of atherosclerotic plaques in mouse thoracic aortas. To unlock the full potential of LSFM and whole-mount immunohistochemistry we combine this methodology with image quantification by deep learning. Deep learning has in recent years revolutionized automated image classification and object detection methods^20–23^. The field of radiology has a long history of applying deep learning^24–26^, in recent years the technology has also been combined with LSFM to enhance the segmentation of labelled structures^13,27,28^. Here we apply deep learning in two steps of our workflow: we divide the aorta into different anatomical segments and in each of them quantify individual atherosclerotic plaque objects and their volume. The deep learning models we apply are 2D and 3D U-nets^29,30^, which are widely used network architectures in biomedical image segmentation. The developed methodology enables precise automated quantification of plaque formation and morphology in mouse models of atherosclerosis.

The code underlying our deep learning models is publicly available at https://github.com/novonordisk-research/aorta_3D.

## Results

### Total plaque volume detection by deep learning

We have developed a method to evaluate plaque burden and composition using 3D imaging with light-sheet fluorescence microscopy and a deep learning model for automatic plaque quantification and segmentation. Training data for the plaque detection model were generated on aortas from 12 ApoE−/− and LDLr−/− mice kept on a western diet for 11–15 weeks. The aortas were cleared using an ECi clearing protocol^31^ or stained and cleared using the iDISCO + protocol^32^ and imaged with a Lavision light-sheet ultramicroscope II (Miltenyi Biotec GmbH, Bergisch Gladbach, Germany). Atherosclerotic plaque was manually annotated on 640 2D optical cross-section images.

**Figure 1:**
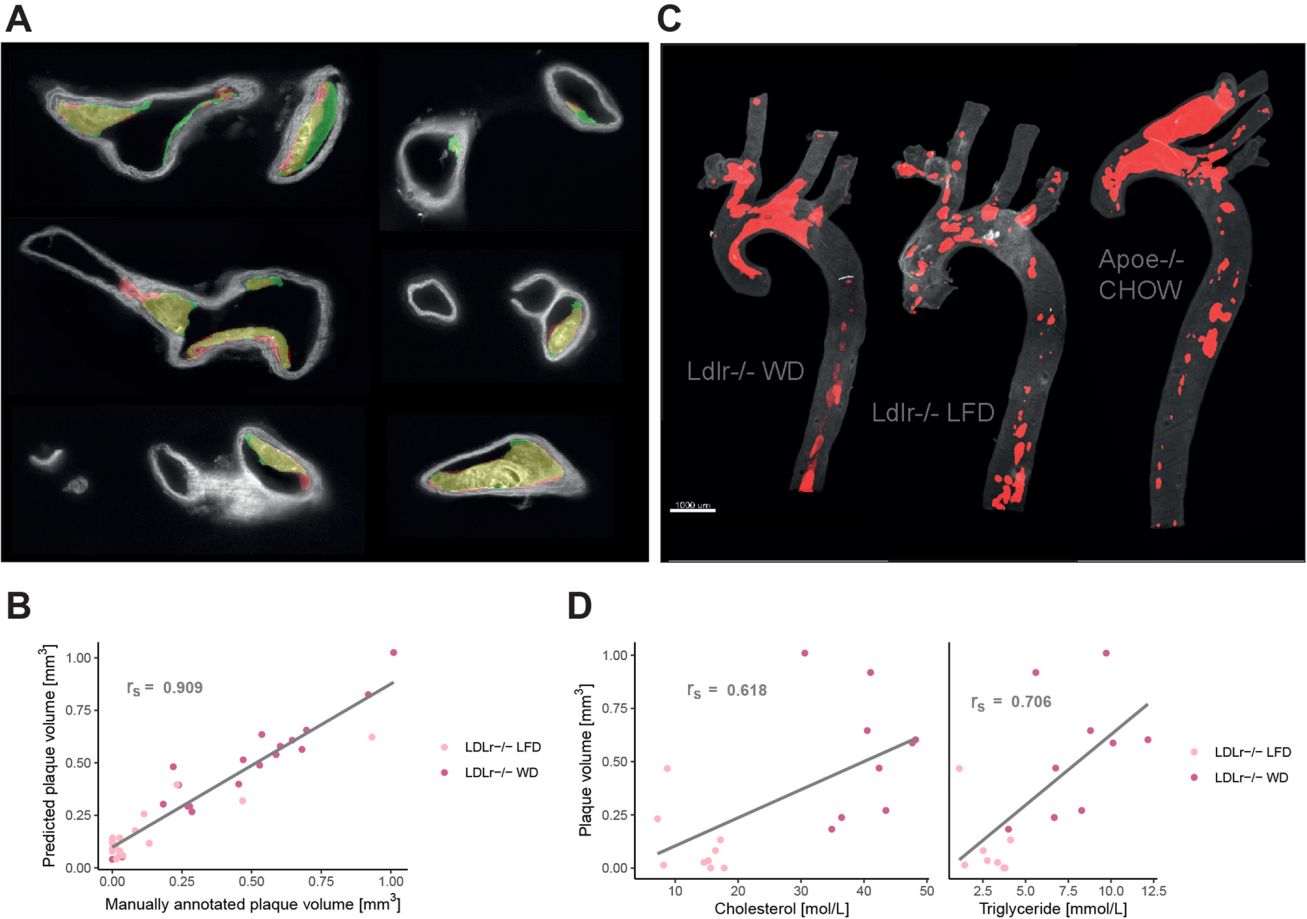
Plaque identification based on tissue autofluorescence. (**A**) Images of the aorta with annotated plaques shown in green and plaque predicted by the deep learning model in red (overlap in yellow). All images are part of the test set and derived from two aortas of Apoe−/− mice on western diet. (**B**) Spearman correlation (r_s_ = 0.909, *p* value < 2.2e−16) between manually annotated plaque volume and plaque volume predicted by the deep learning model. Regression lines added for visualization purposes. (**C**) Plaque predictions by the model in 3D reconstructed aortas. (**D**) Spearman correlation of plaque volume with plasma cholesterol (r_s_ = 0.618, *p* value = 0.007) and triglyceride (r_s_ = 0.706, *p* value = 0.002). Regression lines are added for visualization purposes.

To predict plaque we trained a 2D U-net^29^, a widely used deep learning model within biomedical image segmentation. The model performed well in predicting plaque objects (Fig. 1A), with a dice coefficient of 0.74 on the test set. 3D plaque volume (Fig. 1B) was quantified by applying the model to each image in an image stack obtained from the aortas.

To ensure our model provides reliable plaque volume predictions, we acquired data from another 37 aortas of LDLr−/− mice; 19 on low fat diet and 18 on western diet. Manual annotations of plaque were performed on every 5th image, allowing the calculation of total plaque volume in mm^3^ using the image processing software Imaris (Bitplane version 7.3.1, Oxford Instruments). Plaque segmentation by deep learning on these samples was highly correlated to manual annotations (r_s_ = 0.909, *p* value < 2.2e−16), which verified that the algorithm provides reliable plaque volume estimates (Fig. 1C).

Plaque burden in our models was correlated to in vivo levels of cholesterol (r_s_ = 0.618, *p* value = 0.007) and triglycerides (r_s_ = 0.706, *p* value = 0.002) (Fig. 1D). The mechanism of plaque formation is initiated by lipid deposition in the vessel wall, mainly driven by LDL cholesterol deposition (in humans) and VLDL cholesterol deposition (in atherosclerosis prone mouse models)^3,33–36^. Therefore, the positive correlations between plaque burden and lipids support the validity of the method of plaque quantification used here. Together these results demonstrate that deep learning can accurately identify atherosclerotic plaques in the aorta and neighbouring branches based on the autofluorescent nature of the tissue.

### Plaque distribution and composition

An additional advantage of a deep learning model able to detect and quantify plaque volume in aortic samples is the possibility to simultaneously investigate the distribution and composition of atherosclerotic plaque. For this we examined ApoE−/− mice, which were either fed a chow or western diet for 14 weeks (10 animals per group). Upon dissection, whole mount immunostaining and clearing of the aorta samples was performed using the established iDISCO + protocol^8^. Aortas were subsequently imaged with a Lavision light-sheet ultramicroscope II (Miltenyi Biotec GmbH, Bergisch Gladbach, Germany).

The model described above was trained and validated on images obtained with a microscope using tile based acquisition, while the new study in ApoE−/− mice was imaged using dynamic acquisition. Because the model did not generalize to the new images, we manually annotated a limited amount of these images to fine tune our model (dice coefficient 0.849). Using these additional annotations, we showed that 25–50 annotated images were sufficient to fine tune the model to a new microscope/acquisition mode (Supplemental Fig. 1A).

**Figure 2:**
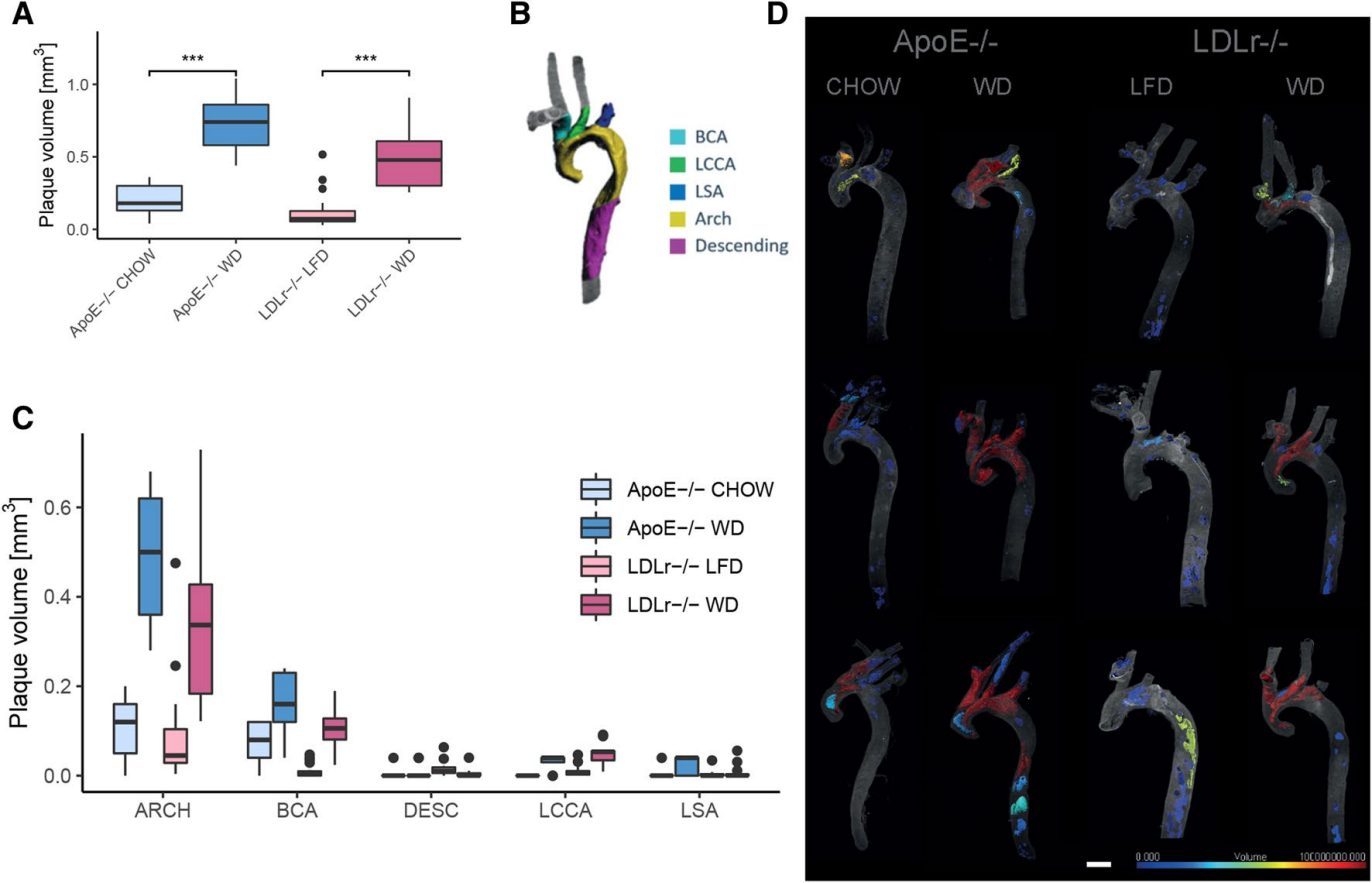
Plaque burden throughout the aorta and branching vessels. (**A**) Total plaque volume in ApoE−/− and LDLr−/− mice on different diets. Plaque volume is significantly increased in mice on western diet (Man-Whitney test, ApoE−/− *p* value = 0.0002, LDLr−/− *p* value = 1.15e-7). (**B**) Division of the aorta into 5 anatomical structures. (**C**) Plaque volume per anatomical structure. (**D**) Visualization of the sizes of individual plaque objects (scale bar 1000 μm).

With the fine-tuned model, we compared the total plaque volume found in the different diet groups of the 20 ApoE−/− mice to the previously described 37 LDLr−/− mice. In line with previous observations^37,38^, we found an overall higher amount of plaque in ApoE−/− mice compared with LDLr−/− mice (*p* value = 0.03), while plaque burden was increased in animals fed western diet in both mouse strains (ApoE−/− *p* value = 0.0002, LDLr−/− *p* value = 1.15e−7) (Fig. 2A).

Due to diverse hemodynamic factors (low or oscillating shear stress) atherosclerotic plaques are known to develop preferentially at arterial branching and bifurcation sites^39^. However, precise quantification of plaque burden in arterial branches and comparison of the two most widely used atherosclerosis prone mouse models have not been reported previously. With our data, we could now quantify plaque burden in different anatomical sections of the aorta and connected branches. For this, we manually segmented our aorta tissue into the following structures: descending aorta (DESC), aortic arch (ARCH), brachiocephalic artery (BCA), left subclavian artery (LSA) and left common carotid artery (LCCA) shown in Fig. 2B. Additionally we trained a 3D U-net to automate the anatomical segmentation of aortas (Supplemental Fig. 1B).

The plaque volume in different sections of the aorta revealed that the plaque burden was highest in the aortic arch and brachiocephalic artery (Fig. 2C, D). This effect was significant in both, ApoE−/− mice (FDR corrected *p* values <  = 0.01) and LDLr−/− mice (FDR corrected *p* values <  = 3.81e-5) (Supplemental Tables 3, 4).

**Figure 3:**
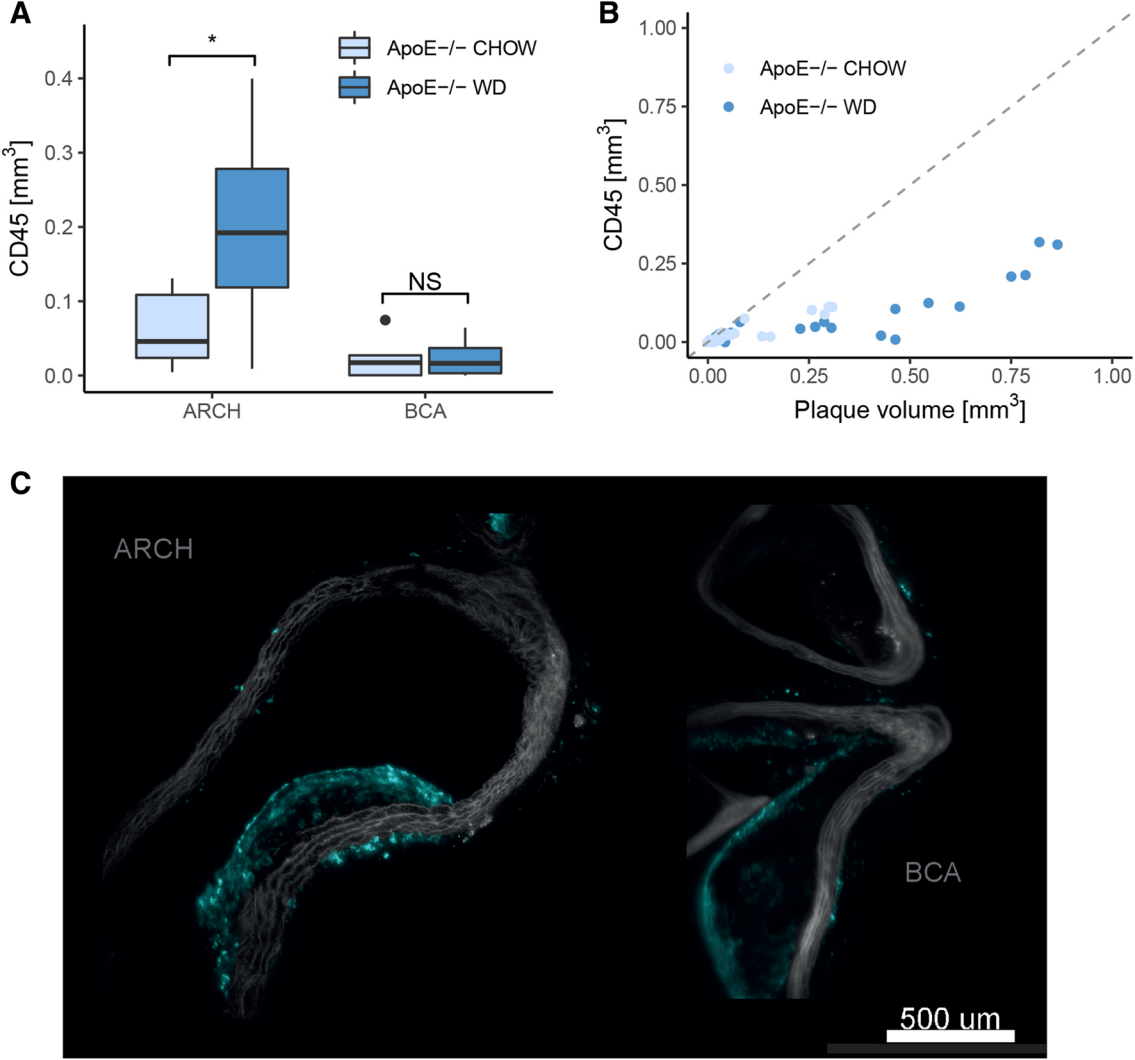
Plaque composition in terms of CD45 signal. (**A**) Immune cell marker CD45 signal in ApoE−/− mice in different vessel compartments. CD45 volume is increased in the aortic arch of ApoE−/− mice on western diet (FDR corrected *p* value = 0.014), but not in the BCA branch (FDR corrected *p* value = 0.95). (**B**) Composition of individual plaque objects in terms of CD45 signal and total volume. (**C**) Optical cross section images reveal that CD45 is only found in the periphery of larger plaques. Left image: aortic arch, right image: BCA. Both images are from Apoe−/− mice fed western diet.

Furthermore, we applied an anti-CD45 immunostaining to the samples to study plaque composition. Here we found that CD45 signal was increased in the aortic arch of ApoE−/− mice on western diet (*p* value = 0.014, Fig. 3A). In the brachiocephalic artery however, no difference in CD45 signal between chow and western diet groups was observed (*p* value = 0.95). This observation contrasts with the increase in plaque volume observed in the brachiocephalic artery for animals fed a western diet (Fig. 2C).

When investigating the composition of individual plaque objects (Fig. 3B), we found that CD45 levels remain mostly constant while plaque volume increases. Looking into optical cross section images (Fig. 3C) we found that some smaller plaques are heavily infiltrated by CD45 stain, while larger plaques only accumulate CD45 in the periphery. This is in line with published knowledge on the progression of atherosclerotic plaque^40,41^. As plaque matures and develops acellular necrotic cores, leukocyte presence is gradually more restricted to the periphery. These data demonstrate 3D imaging can be used for visualizing and quantifying subtle differences in plaque maturation over the arterial tree.

## Discussion

Pre-clinical models of atherosclerosis can help us understand the dynamics and mechanisms underlying plaque development and evolution. High variability in plaque formation along the vascular tree makes it difficult to acquire a holistic quantitative view of the disease and complicates the study of new therapeutics.

A traditional way of evaluating atherosclerotic plaque burden is by the *en face* method. Here the thoracic aorta is dissected, opened up longitudinally (optionally stained with oil red O or Sudan IV) and imaged by a microscope holding a camera. The plaque area is then determined as a percentage of the intimal surface area^7^. The clearing step in the 3D staining protocol applied here, prevents the possibility of obtaining *en face* values from the same specimen. However, Lloyd et al.^42^ have previously shown that plaque volume measured by microCT and *en face* values are highly correlated. This indicates that 3D light sheet imaging and quantification can replace *en face* values, while providing detailed information regarding plaque composition and most importantly on their volume distribution in the vascular tree that is lost in traditional 2D analyses.

Using light-sheet microscopy to study plaque formation in different blood vessels has very recently been suggested by Becher et al.^19^. Optical cross-sections obtained by LSFM were shown to correlate well with traditional histological stained tissue sections. However, the variation between samples and in the geometry of individual plaques observed by Becher et al. as well as in this study suggests that plaque burden may be more accurately measured in 3D. Until now dedicated image analysis was lacking for quantitative assessment of plaque along the vasculature and every sample had to be analyzed separately, which is both time consuming and may lead to increased variability. The hereby implemented deep learning-based image analysis can automatically be applied to large sample sizes, thereby minimizing hands-on time and enabling accurate comparison of disease progression.

One limitation of LSFM is the limited amount of stains that can be applied to highlight different components of a sample. By using a deep learning approach to identify plaque based only on the autofluorescence signal, we circumvent the need to stain for endothelial layers and nuclei, thus freeing up wavelengths for additional stains. We have here shown the possibility of studying inflammation associated with atherosclerosis, but similar methodology can also be used to automatically quantify the expression of any target protein or cellular marker inside the plaque.

Cole et al.^43^ did not find any difference in total CD45 + cells in ApoE−/− mice on CHOW and western diet using flow cytometry. However, this included resident immune cells in the vascular wall and in the adventitia, while we only quantify CD45 + cells in plaque. Another flow cytometry study by Winkels et al.^44^ found an increase of macrophages in plaque acquired by laser capture microdissection. Here we observed increased CD45 signal in animals on western diet in the aortic arch but not BCA.

Plaque burden in the aortic arch and BCA branch have been measured before using various approaches^7,19,39,42^. Our study provides the first quantitative comparison of plaque distribution in the whole aortic tree. Sample sizes of 10 animals per diet group in ApoE−/− mice are rather small and should be increased in future studies. The developed methodology will enable comparison of plaque changes for future drug discovery studies aimed at reducing plaque burden.

The deep learning model we trained for plaque identification is a U-net, one of the first fully convolutional architectures suggested specifically for biomedical image segmentation^29^. Some variations and extensions have been suggested to the classical U-net architecture^45–49^, benchmarking these in future work could potentially result in slightly increased model performance. One limitation of this approach is the need to fine-tune the model to new imaging equipment, creating a need for specialized computational skills when adapting this approach in a new laboratory. We have made the underlying code available to facilitate and speed up this process.

For the anatomical segmentation of aorta samples we propose a 3D U-net^30^, which in contrast to an image registration process does not require the sample to be imaged in a certain orientation, thus further automating the process.

Concluding, we have presented an approach combining LSFM and deep learning models for analysing atherosclerotic plaque volume and composition in different anatomical sections of the aorta. Using the here developed technology we provide a comprehensive and quantitative overview of plaques in the common mouse models of atherosclerosis. Our quantitative 3D imaging technique enables simultaneous volumetric analysis of plaques and their composition (e.g. inflammatory cells) in the aorta and the main arterial branches. The approach is applicable for large sample sizes and if there is a further need for histological analysis on parts of the tissue, aortas can be rehydrated, embedded in paraffin and sectioned after LSFM imaging. The here presented imaging and analysis strategy can also be adapted to other vessels, and we provide the code underlying our models for easy implementation.

## Materials and methods

### Mice

Male LDLr−/− mice were purchased from Jackson Laboratories and female ApoE−/− mice (C57bl background) from Charles River. All animals were single housed at Novo Nordisk A/S in temperature (22 ± 2 °C) and humidity (50 ± 20%) controlled rooms with a 12/12 h circadian rhythm. The care and use of mice in these studies were conducted according to national legislation in Denmark and all experiments were approved by the Animals Inspectorate (Dyreforsøgstilsynet) governed by the Danish Ministry of Food and Agriculture. Age and body weight of the mice used in the experiments are listed in Table 1. All mice were acclimated for 2 weeks on standard chow diet (Altromin 1324, Brogaarden, Denmark) before being assigned to a diet group. All mice in study 1 and 2 were all fed a western diet for 9–14 weeks before the mice were euthanized and aortas collected. Study 3 and 4 mice were allocated to different diet groups based on body weight. In study 3, LDLr−/− mice were fed a western diet (n = 20, D12079B, Research Diet Inc, New Brunswick, New Jersey, USA) or a low fat diet (n = 21, D12450B, Research Diet Inc, New Brunswick, New Jersey, USA) for 14 weeks. In study 4, ApoE−/− mice were kept on the standard chow (n = 10) or fed the western diet (n = 10) for 14 weeks.

**Table 1:** Overview of studies included for training, validation and testing of the plaque model.

Study	Genotype	Age at arrival (weeks)	Gender	n	Weeks on western diet	Used for
1	ApoE−/−	6–8	Female	4	14	Model training, validation and testing
2	ApoE−/−	8–10	Female	6	9	Model training, validation and testing
3	LDLr−/−	8–18	Male	41	14	Model training and validation (n=4), testing (n=37)
4	ApoE−/−	7–10	Female	20	14	Model fine tuning

### Lipid profile

Retroorbital blood samples were collected at termination in K3-EDTA-coated tubes. Plasma (centrifuged at 4 °C; 3500 rpm; 10 min) were used for triglyceride and total cholesterol analyses (Cobas 6000 Multianalyser, Roche Diagnostics, Rotkreuz ZG, Switzerland, Cat#20767107322 and Cat#3039773190).

### Aorta dissection

At study termination, mice were anesthetized with fentanyl/fluanisone and midazolam (subcutaneous 7.5 ml/kg), followed by cardiac perfusion with 10 mL of ice-cold saline. The aorta was excised at the aortic root (2 mm from the heart) and at the 8th rib.3–4 mm of the 3 large branches at the aortic arch (brachiocephalic, left common carotid and left subclavian arteries) were left on the specimen. The periaortic adipose tissue was carefully removed and the aorta with neighbouring branches was fixed in 10% neutral buffered formalin for 24 h.

### Immunohistochemistry and clearing

Aortas of study 1 and 2 were dehydrated at room temperature in increasing concentrations (20%, 40%, 60%, 80%, 100%) of ethanol at 1 h per step followed by overnight incubation in 100% ethanol. Next day, the aortas were transferred to ECi until transparent. Aortas from study 3 were prepared using the previously described iDISCO protocol^8^ with slight modifications. Ethanol was used for dehydration and ECi was used for clearing. Aortas from study 4 were also prepared using the iDISCO procedure, whole-mount immunostained with anti-CD45 primary antibody (BD Pharmingen, AB_2174426, 1:200) and donkey anti-rat IgG secondary antibody (Cy3, Jackson ImmunoResearch, AB_2340667, 1:1000). Methanol was used for dehydration and DBE for clearing. A full description of all procedures can be found in Supplementary materials under “Immunohistochemistry and clearing”.

### Light sheet fluorescence microscopy

Aortas in study 1–3 were imaged using a Lavision light-sheet ultramicroscope II (Miltenyi Biotec GmbH, Bergisch Gladbach, Germany) with Zyla 4.2P-CL10 sCMOS camera (Andor Technology, Belfast, United Kingdom), SuperK EXTREME supercontinuum white-light laser EXR-15 (NKT photonics, Birkerød, Denmark) and MV PLAPO 2XC (Olympus, Tokyo, Japan) objective. Samples were mounted directly onto the sample holder with Loctite 401 Universal glue (Henkel) and imaged in an ECi filled chamber. Imspector microscope controller software (v7) was used (Miltenyi Biotec GmbH, Bergisch Gladbach, Germany). Samples were illuminated by three light sheets from left side only. Horizontal images were acquired as 4 tiles at 1 × magnification (2 × total magnification) with an exposure time of 100 ms in a z-stack at 8 µm intervals. All tiff images were generated in 16-bit. Autofluorescence images were captured at 545 ± 30 nm excitation wavelength and 620 ± 60 nm emission wavelength.

Aortas from ApoE−/− mice in study 4 were imaged using a similar setup as study 1–3 with the following changes: Samples, which were embedded into agarose, were mounted on a transparent silicone sample holder with neutral silicone gel (Danalim) and imaged in a DBE filled chamber. Samples were illuminated by laser light from both sides. Horizontal images were acquired at 0.63 × magnification (1.2 × total magnification) with an exposure time of 227 ms in a z-stack at 7 µm intervals. Horizontal focusing was captured in 9 planes with contrast adaptive merging of individual raw images (dynamic acquisition). For high magnification images 6.3 × magnification (12.6 × total magnification) was used with an exposure time of 307 ms. CD45 staining was imaged at 560 ± 20 nm (excitation) and 650 ± 25 nm (emission) wavelength. Autofluorescence images were captured at 630 ± 15 nm excitation wavelength and 680 ± 15 nm emission wavelength.

### Manual plaque volume measurement

In image stacks from 37 of the 41 aortas in study 3 plaque was annotated in every 5th image (corresponding to every 40 μm). Based on the annotations, manual plaque volume was calculated in Imaris (Bitplane version 7.3.1).

### Plaque segmentation model

ITK Snap (version 3.6.0) was used to manually annotate plaques on optical 2D cross section images of aortas. Annotations were saved as binary mask.

A total of 640 images were annotated from 14 aortas (10 Apoe−/− from study 1 and 2 and 4 Ldlr−/− mice from study 3). The training set consisted of 504 annotated images derived from 6 aortas of Apoe−/− mice and 4 aortas of Ldlr−/− mice. The validation and test set consisted of 63 and 73 images respectively, each derived from 2 aortas of Apoe−/− mice. Images were downsized to 608 × 480 pixels and subsequently normalized using adaptive histogram equalization.

A 2D U-net^29^ was trained to segment atherosclerotic plaque. Neural network training was implemented in Python using the keras library^50^. The learning rate was set to 0.0001 and the Adam optimizer^51^ was applied. The networks prediction error was measured as 1-dice coefficient, where the dice coefficient is defined as d = (2*XY + 1)/(X + Y + 1), where X = prediction and Y = target. Networks were trained for a total of 500 epochs using early stopping. Data augmentation, in the form of skews, rotations, flips, zoom and random distortions (30% probability each) was applied during training.

### Fine tuning for dynamic acquisition

The model described above, trained on images obtained by tile-based acquisition, was fine tuned to perform well on images obtained using dynamic acquisition. For this 253 images of aortas from 20 ApoE−/− mice (experiment 2) were annotated. Images were downsized to 608 × 480 pixels and adaptive histogram equalization was applied. The data were randomly split into a training set of 151 images, a validation set of 63 images and a test set of 39 images.

The above described U-net was then fine tuned on these data for 300 epochs using early stopping. Additionally, we subsampled from the training set (25, 50, and 100 images, sampled 3 times each) and fine tuned several models as described above to investigate how many annotations were necessary to adapt the model to a new imaging mode or different microscope.

### Aorta anatomy labelling

An ensemble aorta atlas was created by manual annotation of eight representative aortas in Imaris (Bitplane version 9.2.0). The atlas includes five parts: descending aorta, aortic arch, left subclavian artery (LSA), left common carotid artery (LCCA), and the brachiocephalic artery (BCA). The segmentation of a new sample was obtained by performing image registration with all eight atlas samples and choosing the best result based on the dice score between a binary version of the sample and the atlases. Preprocessing for registration included down-sampling (to 300 × 260 × 190 pixels) and creation of a filled binary volume which was then encoded by the distance to a background voxel. Registration included global rigid and affine alignment followed by local b-spline alignment. After registration samples were post-processed to fill holes, remove parts of the atlas outside the aorta autofluorescence image and label pixels within the aorta not covered by the atlas according to the nearest neighbour.

Subsequently the aorta volume images were binarized and downsized to 64 × 64 × 64 × 1 pixels. The corresponding atlas volumes were downsized to 64 × 64 × 64 × 6 (accounting for all 5 labelled sections of the aortic tree and the remaining unlabelled part of the aorta). A total of 62 aortas (20 from Apoe−/− mice and 42 from Ldlr−/− mice) were used in the process. 3D U-nets were trained to predict the atlas labels based on the binary volume image of the aorta. In total 4 networks were trained, for each we left out 2 aortas (one Apoe−/− and one Ldlr−/−), the rest of the aortas were rotated by 90 degrees around all three axis to augment the data set. Subsequently 10% of the aortas were randomly selected to represent the validation set, while the rest were used for model training. The networks were trained for 300 epochs using early stopping. The network prediction error was measured as dice coefficient separately for each labelled aorta compartment and subsequently averaged. Weights were updated based on the Adam optimizer using a learning rate of 0.00001. To evaluate performance, network predictions were up-scaled to 300 × 260 × 190 pixels and post processed to fill holes in predictions, remove predictions outside the aorta, label unlabelled parts of the aorta or parts with overlapping predictions according to the nearest neighbour class. Performance was finally calculated as dice coefficient for all compartments between the original atlas and the post-processed predictions.

Code and models are available here: https://github.com/novonordisk-research/aorta_3D.

### Statistical analysis

Plaque volumes measured in this study are not normally distributed but skewed, due to the low amount of plaque in animals on LFD or CHOW diet. Therefore nonparametric statistical tests were applied. To test for correlation between manually annotated plaque volume and plaque volume predicted by the deep learning model we calculated Spearman’s r_S_. The same procedure was applied to test for correlation between blood lipids and plaque burden. For visualization purposes a linear model was fit to the data.

The Man-Whitney test was applied to test for differences in plaque burden between different diet groups as well as mouse models on the same diet. To compare plaque burden in different anatomical locations within the aorta, the paired-samples Mann Whitney test was used, followed by false discovery rate (FDR) correction of *p* values for multiple testing.

The Man-Whitney test with subsequent FDR correction for multiple testing was applied to test for differences in CD45 volume within plaque in the BCA or aortic arch in ApoE−/− mice on chow diet compared to western diet.

*p* values < 0.05 were considered statistically significant. All statistical tests were performed in R version 3.5.1.

## Supplementary information

Supplementary Information.

## Data Availability

Code and models are available here: https://github.com/novonordisk-research/aorta_3D.

## References

[1] Roth GA (2017). Global, regional, and national burden of cardiovascular diseases for 10 causes, 1990 to 2015. J. Am. Coll. Cardiol..

[2] Bennett MR, Sinha S, Owens GK (2016). Vascular smooth muscle cells in atherosclerosis. Circ. Res..

[3] Basatemur GL, Jørgensen HF, Clarke MCH, Bennett MR, Mallat Z (2019). Vascular smooth muscle cells in atherosclerosis. Nat. Rev. Cardiol..

[4] Libby P, Ridker PM, Hansson GK (2011). Progress and challenges in translating the biology of atherosclerosis. Nature.

[5] Epah J (2018). 3D imaging and quantitative analysis of vascular networks: a comparison of ultramicroscopy and micro-computed tomography. Theranostics.

[6] Vågberg W, Persson J, Szekely L, Hertz HM (2018). Cellular-resolution 3D virtual histology of human coronary arteries using x-ray phase tomography. Sci. Rep..

[7] Centa M, Ketelhuth DFJ, Malin S, Gisterå A (2019). Quantification of atherosclerosis in mice. J. Vis. Exp..

[8] Renier N (2014). iDISCO: a simple, rapid method to immunolabel large tissue samples for volume imaging. Cell.

[9] Di Giovanna AP (2018). Whole-brain vasculature reconstruction at the single capillary level. Sci. Rep..

[10] Mano T (2018). Whole-brain analysis of cells and circuits by tissue clearing and light-sheet microscopy. J. Neurosci..

[11] Soderblom C (2015). 3D imaging of axons in transparent spinal cords from rodents and nonhuman primates. eNeuro.

[12] Cai R (2019). Panoptic imaging of transparent mice reveals whole-body neuronal projections and skull–meninges connections. Nat. Neurosci..

[13] Todorov, M. I. *et al.* Automated analysis of whole brain vasculature using machine learning. *bioRxiv* 613257. 10.1101/613257. (2019).

[14] Pan, C. *et al.* Deep learning reveals cancer metastasis and therapeutic antibody targeting in whole body. *bioRxiv* 541862. 10.1101/541862. (2019). 10.1016/j.cell.2019.11.013 PMC7591821 31835038

[15] Zhao, S. *et al.* Cellular and molecular probing of intact transparent human organs. *bioRxiv* 643908. 10.1101/643908. (2019).

[16] Fei P (2016). Cardiac light-sheet fluorescent microscopy for multi-scale and rapid imaging of architecture and function. Sci. Rep..

[17] Kräker K (2018). 54. Cardiac small vessel imaging by light sheet microscopy and micro CT—discovering the missing link between preeclampsia and higher risk for further cardiovascular disease. Pregnancy Hypertension.

[18] Ding Y (2018). Multiscale light-sheet for rapid imaging of cardiopulmonary system. JCI Insight.

[19] Becher T (2020). Three-dimensional imaging provides detailed atherosclerotic plaque morphology and reveals angiogenesis after carotid artery ligation. Circ. Res..

[20] LeCun Y, Bengio Y, Hinton G (2015). Deep learning. Nature.

[21] Esteva A (2017). Dermatologist-level classification of skin cancer with deep neural networks. Nature.

[22] Poplin R (2018). Prediction of cardiovascular risk factors from retinal fundus photographs via deep learning. Nat. Biomed. Eng..

[23] Coudray N (2018). Classification and mutation prediction from non-small cell lung cancer histopathology images using deep learning. Nat. Med..

[24] Chen (2020). Deep learning for cardiac image segmentation: a review. Front. Cardiovasc. Med..

[25] Litjens G (2019). State-of-the-art deep learning in cardiovascular image analysis. JACC Cardiovasc. Imaging.

[26] Siegersma KR (2019). Artificial intelligence in cardiovascular imaging: state of the art and implications for the imaging cardiologist. Neth. Heart J..

[27] Pan C (2019). Deep learning reveals cancer metastasis and therapeutic antibody targeting in the entire body. Cell.

[28] Roostalu U (2019). Quantitative whole-brain 3D imaging of tyrosine hydroxylase-labeled neuron architecture in the mouse MPTP model of Parkinson’s disease. Dis. Model. Mech..

[29] Ronneberger, O., Fischer, P. & Brox, T. U-Net: convolutional networks for biomedical image segmentation (2015). arXiv:1505.04597v1.

[30] Çiçek, Ö., Abdulkadir, A., Lienkamp, S. S., Brox, T. & Ronneberger, O. 3D U-Net: learning dense volumetric segmentation from sparse annotation. (2016). arXiv:1606.06650v1.

[31] Klingberg A (2017). Fully automated evaluation of total glomerular number and capillary tuft size in nephritic kidneys using lightsheet microscopy. J. Am. Soc. Nephrol..

[32] Renier N (2016). Mapping of brain activity by automated volume analysis of immediate early genes. Cell.

[33] Ference BA (2017). Low-density lipoproteins cause atherosclerotic cardiovascular disease. 1. Evidence from genetic, epidemiologic, and clinical studies. A consensus statement from the European Atherosclerosis Society Consensus Panel. Eur. Heart J..

[34] Cheng JM (2015). Plasma concentrations of molecular lipid species in relation to coronary plaque characteristics and cardiovascular outcome: results of the ATHEROREMO-IVUS study. Atherosclerosis.

[35] VanderLaan PA, Reardon CA, Thisted RA, Getz GS (2009). VLDL best predicts aortic root atherosclerosis in LDL receptor deficient mice. J. Lipid Res..

[36] Knouff C (1999). Apo E structure determines VLDL clearance and atherosclerosis risk in mice. J. Clin. Invest..

[37] Daugherty A (2017). Recommendation on design, execution, and reporting of animal atherosclerosis studies: a scientific statement from the American Heart Association. Circ. Res..

[38] Rakipovski G (2018). The GLP-1 analogs liraglutide and semaglutide reduce atherosclerosis in ApoE−/− and LDLr−/− mice by a mechanism that includes inflammatory pathways. JACC Basic Transl. Sci..

[39] Cheng C (2006). Atherosclerotic lesion size and vulnerability are determined by patterns of fluid shear stress. Circulation.

[40] Kasikara C, Doran AC, Cai B, Tabas I (2018). The role of non-resolving inflammation in atherosclerosis. J. Clin. Invest..

[41] Hansson GK, Libby P (2006). The immune response in atherosclerosis: a double-edged sword. Nat. Rev. Immunol..

[42] Lloyd DJ (2011). A volumetric method for quantifying atherosclerosis in mice by using microCT: comparison to en face. PLoS ONE.

[43] Cole JE (2018). Immune cell census in murine atherosclerosis: cytometry by time of flight illuminates vascular myeloid cell diversity. Cardiovasc. Res..

[44] Winkels H (2018). Atlas of the immune cell repertoire in mouse atherosclerosis defined by single-cell RNA-sequencing and mass cytometry. Circ. Res..

[45] Jegou, S., Drozdzal, M., Vazquez, D., Romero, A. & Bengio, Y. The One Hundred Layers Tiramisu: Fully Convolutional DenseNets for Semantic Segmentation. In *2017 IEEE Conference on Computer Vision and Pattern Recognition Workshops (CVPRW)*. 10.1109/cvprw.2017.156. (2017).

[46] Chen L-C, Papandreou G, Kokkinos I, Murphy K, Yuille AL (2018). DeepLab: semantic image segmentation with deep convolutional nets, atrous convolution, and fully connected CRFs. IEEE Trans. Pattern Anal. Mach. Intell..

[47] Ulku, I. & Akagunduz, E. A survey on deep learning-based architectures for semantic segmentation on 2D images. (2019). arXiv:1912.10230v2.

[48] Garcia-Garcia A (2018). A survey on deep learning techniques for image and video semantic segmentation. Applied Soft Computing.

[49] Minaee, S. *et al.* Image segmentation using deep learning: a survey. (2020). arXiv:2001.05566v5. 10.1109/TPAMI.2021.3059968 33596172

[50] keras-team. keras-team/keras. *GitHub*. https://github.com/keras-team/keras.

[51] Kingma, D. P. & Ba, J. Adam: a method for stochastic optimization (2014). arXiv:1412.6980v9.

