# Supplementary material for: Deep learning reveals 3D atherosclerotic plaque distribution and composition
Source: Sci Rep. 2020 Dec 9;10:21523. doi: 10.1038/s41598-020-78632-4 (PMC7726562; doi:10.1038/s41598-020-78632-4)
Supplement: Supplementary file 1 — Supplementary Information. [file 41598_2020_78632_MOESM1_ESM.pdf]

Deep Learning reveals 3D atherosclerotic plaque distribution and composition

Vanessa Isabell Jurtz[1,*], Grethe Skovbjerg[1,*], Casper Gravesen Salinas[2], Urmas Roostalu[2], Louise Pedersen[1,3], Jacob Hecksher-Sørensen[2], Bidda Rolin[1,#], Michael Nyberg[1], Martijn van de Bunt[1], Camilla Ingvorsen[1]

1. Novo Nordisk A/S, 2760 Maaloev, Denmark
2. Gubra, 2970 Hoersholm, Denmark
3. University of Copenhagen, 1017 Copenhagen, Denmark
# Current address Gubra, 2970 Hoersholm, Denmark
*Communicating author

Correspondence: Vanessa Isabell Jurtz, Novo Nordisk Park, 2760 Maaloev, Denmark. Email: vnij@novonordisk.com

Supplementary materials

Abbreviations

2D	two dimensional
3D	three dimensional
ARCH	aortic arch
BCA	brachiocephalic artery
CHOW	Chow diet
DBE	dibasic ester
DESC	descending aorta
DMSO	dimethyl sulfoxide
ECi	ethyl cinnamate
FDR	false discovery rate
LCCA	left common carotid artery
LFD	low fat diet
LSA	left subclavian artery
LSFM	light-sheet fluorescence microscopy
WD	western diet

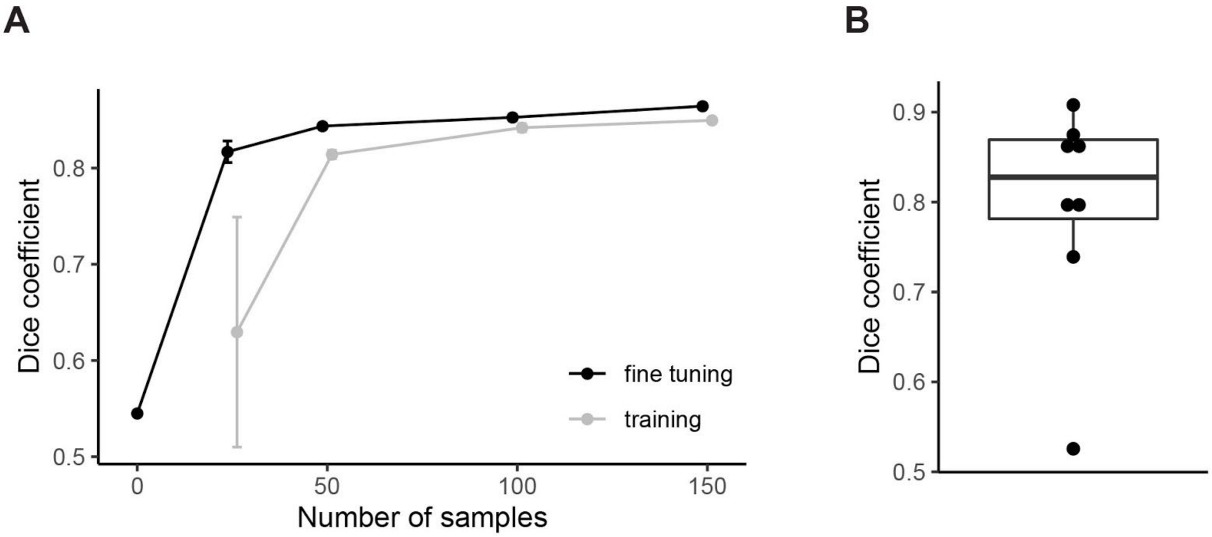

Supplemental Figure 1: A Fine-tuning a U-net trained on images generated through tile acquisition to dynamic acquisition compared to training a U-net from scratch on dynamic acquisition data. Three U-nets were trained on randomly subsampled training sets of 25, 50 and 100 samples. **B** Performance of the 3D U-net segmenting anatomical structures in the aortic tree. A total of 4 models were trained and evaluated here on 2 aortas.

Supplemental table 1: Times in days for conducting a histology study on BCA samples of 100 mice. The study was conducted at Novo Nordisk A/S and the results are not published.

Procedure	Histology of BCA		
		100 samples (4 blocks)	
		total	hands-on
Trim + agar embedding + paraffin embedding		1,5	1,5
Sectioning (~350 sections per block)		2	2
Staining	IHC for macrophages on every 10th section	2	2
Scanning		4	1
Image analysis		4	1
Data sorting and calculations		2	2
**Sum**		**15,5**	**9,5**

Supplemental table 2: Estimated time for conducting histology and LSFM studies on different sample sizes. Histology time estimates are extrapolated from the data shown in supplemental table 1.

Procedure	Histology[1]						
		25 samples (8 blocks)		50 samples (16 blocks)		100 samples (32 blocks)	
		total	hands-on	total	hands-on	total	hands-on
Trim + agar embedding + paraffin embedding		4	4	5	5	6	6
Sectioning (~350 sections per block)		4	4	8	8	16	16
Staining	IHC for macrophages on every 10th section	8	8	16	16	32	32
Scanning		8	2	16	4	32	8
Image analysis		8	2	16	4	32	8
Data sorting and calculations		4	4	8	8	16	16
**Sum**		**36**	**24**	**69**	**45**	**134**	**86**
	**LSFM**						
		25 samples		50 samples		100 samples	
		total	hands-on	total	hands-on	total	hands-on
Staining (iDISCO)	Dehydrationa + de-lipidation	1	1	1	1	1	1
	Wash and bleaching	1	0,5	1	0,5	1	0,5
	Rehydration and wash	1	1	1	1	1	1
	Permeabilisation	3	0,25	3	0,25	3	0,25
	Blocking	2	0,25	2	0,25	2	0,25
	Primary antibody	7	0,25	7	0,25	7	0,25
	Wash	1	1	1	1	1	1
	Secondary antibody	7	0,25	7	0,25	7	0,25
	Wash (+ agar embedding)	2	2	2	2	2	2
	Dehydration + de-lipidation + clearning	2	1	2	1	2	1
Scanning		2	0,5	3,5	0,857	6,5	1,625
Image analysis		0,04		0,04		0,04	
**Sum**		**29,04**	**8**	**30,54**	**8,357**	**33,54**	**9,125**

1 Estimates are extrapolated based on experience with studies on BCA only

Supplemental table 3: Comparing plaque volume in different locations within the aorta of ApoE-/- mice on western diet. The table shows p-values obtained by two-sided paired-samples Mann Whitney Wilcoxon test after FDR correction for multiple testing.

	**ARCH**	**BCA**	**DESC**	**LCCA**	**LSA**
**ARCH**		**0.0098**	**0.0098**	**0.0098**	**0.0098**
**BCA**			**0.0098**	**0.0098**	**0.0113**
**DESC**				0.2018	0.1618
**LCCA**					1.0000
**LSA**					

Supplemental table 4: Comparing plaque volume in different locations within the aorta of LDLr-/- mice on western diet. The table shows p-values obtained by two-sided paired-samples Mann Whitney Wilcoxon test after FDR correction for multiple testing.

	**ARCH**	**BCA**	**DESC**	**LCCA**	**LSA**
**ARCH**		**2.18e-5**	**2.18e-5**	**2.18e-5**	**2.18e-5**
**BCA**			**2.18e-5**	**3.81e-5**	**2.18e-5**
**DESC**				**2.18e-5**	0.89
**LCCA**					**4.24e-4**
**LSA**					

Major Resources Table

Animals (in vivo studies)

Species	Vendor or Source	Background Strain	Sex	Persistent ID / URL
mouse	The Jackson Laboratory	B6.129S7-Ldlrtm1 Her/J	f	Cat# JAX:002207<br>RRID:IMSR_JAX:002207
mouse	Charles River	B6.129P2-*Apoe*^{tm1} *Unc/J*	m	Cat#JAX:002052<br>RRID:IMSR_JAX:002052

Antibodies

Target antigen	Vendor or Source	Catalog #	Working concentration	Lot # (preferred but not required)	Persistent ID / URL
Rat monoclonal anti-mouse CD45	BD Pharmingen	550539	0.31 ug/mL	Lot#8018762	RRID:AB_2174426
Cy3-AffiniPure Donkey Anti-Rat IgG	Jackson ImmunoResearch	712-165-153	1.5 ug/mL		RRID:AB_2340667
Rabbit anti-CD68	Abcam	ab125212	0,08 ug/mL	GR300628	AB_10975465

Donkey anti-rabbit Cy5	Jackson ImmunoResearch	711-175-152	1.5 ug/mL	138336	RRID:AB_2340607

Data & Code Availability

Description	Source / Repository	Persistent ID / URL
Code repository containing the models	This manuscript	https://github.com/novonordisk-research/aorta_3D

Other

Description	Source / Repository	Persistent ID / URL
Ethyl cinnamate (ECi)	Sigma-Aldrich	Cat#112372-100G
Dibenzylether (DBE)	Sigma-Aldrich	Cat#33630-1L
Dichloromethane (DCM)	VWR	Cat#83682.290
Hydrogen peroxide solution (H2O2, 35 wt%)	Acros Organics	Cat#7722-84-1
Glycine	Sigma-Aldrich	Cat#G7126
Agarose	Thermo Fisher	Cat#16520050
Methanol	VWR	Cat#: 20847.318

Ethanol	Plum	E201153-2
Triton X-100	Sigma-Aldrich	Cat#: x100-100M
Dimethyl Sulphoxide (DMSO)	Sigma-Aldrich	Cat#: 41639-500m
Donkey Serum	Jackson Immunoresearch	Cat#: 017-000-121
Heparin	LEO Pharma	Cat#: DH7808
Tween-20	VWR	Cat#: 663684B
Cobas reagent kit triglyceride	Roche	Cat#20767107322
Cobas reagent kit cholesterol	Roche	Cat#: 3039773190
ITK Snap version 3.6.0	ITK Snap	http://itksnap.org SCR_002010
Imaris x64 software version 7.3.1 and 9.2.0	Oxford Instruments	https://imaris.oxinst.com/<br><br>SCR_007370
Imspector Pro software v7	Miltenyi Biotech	https://www.lavisionbiotec.com/

Immunohistochemistry and clearing

ApoE-/- aortas of study 1 and 2 were dehydrated at room temperature in increasing concentrations (20%, 40%, 60%, 80%, 100%) of ethanol at 1 hour per step followed by overnight incubation in 100% ethanol. Next day, the aortas were transferred to ECi until transparent.

Aortas from study 3 were processed in a whole-mount immunohistochemistry protocol, however, only images from the autofluorescence channel were acquired for plaque burden estimate. Samples were dehydrated at room temperature in increasing concentrations (20%, 40%, 60%, 80%, 100%) of ethanol at 1 hour per step. This was followed by bleaching in a 6 % hydrogen peroxide solution in 100% ethanol at 4°C. Tissue was then incubated in decreasing concentrations of ethanol in PBS/0.2% Triton X-100 (80%, 60%, 40%, 20%) followed by 2 washes in PBS/0.2% Triton X-100 (PTx.2), 1 hour per step. Aortas were permeabilized in PBS/0.2% Triton X-100/20% DMSO/0.3M glycine overnight at 37°C. This was followed by blocking in PBS/0.2% Triton X-100/10%DMSO/6% donkey serum overnight at 37°C. Tissues were incubated with rabbit anti-CD68 (Abcam ab125212, 0,08 ug/mL), 2 days at 37°C. The aortas were washed 7 times in PBS/0.2% Tween-20 with 10 ug/ml heparin (PTwH) and incubated with donkey anti-rabbit Cy5, Jackson ImmunoResearch, (711-175-152, 1.5 ug/mL) in PBS/0.2% Tween-20 with 10 ug/ml heparin (PTwH)/3% Donkey Serum overnight at 37°C.

Tissues were washed 6 times and incubated overnight in PTwH at room temperature. Tissues were dehydrated in increasing concentrations (20%, 40%, 60%, 80%, 100%) of methanol at 1 hour per step, followed by overnight incubation in 100% ethanol. Finally, aortas were transferred to ECi for clearing.

Aortas from study 4 were also prepared using a whole-mount immunohistochemistry protocol. Samples were dehydrated at room temperature in increasing concentrations (20%, 40%, 60%, 80%, 100%) of methanol at 1 hour per step. This was followed by overnight incubation in a DCM:methanol (2:1) solution at room temperature. Tissues were washed twice in 100% methanol, followed by overnight incubation in a 6 % hydrogen peroxide solution in 100% methanol at 4°C. Tissue was then incubated in decreasing concentrations of methanol in PBS/0.2% Triton X-100 (80%, 60%, 40%, 20%) followed by 2 incubations in PBS/0.2% Triton X-100 (PTx.2), 1 hour per step. Aortas were permeabilized in PBS/0.2% Triton X-100/20% DMSO/0.3M glycine, 3 days, 37°C. This was followed by blocking in PBS/0.2% Triton X-100/10%DMSO/6% Donkey serum, 2 days, 37°C. Tissues were incubated with rat anti-CD45 (BD Pharmingen, AB_2174426, 1:200) diluted in PTwH/5% DMSO/3% Donkey Serum, 7 days, 37°C. The aortas were washed 7 times in PTwH and incubated with donkey anti-rat IgG (Cy3, Jackson ImmunoResearch, AB_2340667, 1:1000) in PBS/0.2% Tween-20 with 10 ug/ml heparin (PTwH)/3% Donkey Serum, 7 days, 37°C. Tissues were washed 6 times and incubated 3 days

in PTwH at room temperature. After the final wash, the samples were embedded in 1% low melting-point agarose (Thermo Fisher, cat#16520050, dissolved in PBS). Next, the agarose-embedded tissues were dehydrated in increasing concentrations (20%, 40%, 60%, 80%, 100%) of methanol at 1 hour per step, followed by overnight incubation in 100% methanol. The aortas were then incubated for 3 hours in DCM:methanol (2:1), followed by 2 x 15 min in 100% DCM. Finally, aortas were transferred to DBE for clearing
